# An Experimental and Clinical Study of Ergot

**Published:** 1891-09

**Authors:** 


### AN EXPERIMENTAL AND CLINICAL STUDY OF ERGOT.

---

In a series of carefully and elaborately conducted experiments at the Biological Laboratory of John Hopkins University, on the physiological effects of ergot, Dr. John C. Hermeter has arrived at a solution of some of the vexed and antagonistic theories of the action of this drug. He employed various preparations of ergot, and states, "I concluded to resort to the fluid extract of Ergot, but found this as obtained from various sources very variable, both in physical properties and therapeutic efficacy, some specimens having a very offensive odor. In two German preparations of ergotine and one liquid ergotine prepared in Basle, Switzerland, and speciallv recommended by the manufacturer for hypodermic use, a very unpleasant foetid odor, reminding one of decomposed organic matter was noticeable, and the last named used hypodermically proved very irritating, causing an abscess, in a patient suffering from goitre. My attention was at last called to a form of liquid ergotine made in Baltimore, by Sharp & Dohme, which gave evidences of being a standard preparation, both in clinical and experimental application. I have had some of this *ergotole* in my possession for nearly ten months. It has deposited no sediment, has a fresh and pure odor, and is very effective. This ergotine solution, which is the most concentrated liquid preparation of ergot that can be obtained, has since become known under the name of *ergotole.* In the experiments to be described, this form of ergot was used."

He then goes on to describe his experiments made upon rabbits. The objects of the experiments were:

1. To determine whether the contractions of the uterus by ergot is of centric or peripheral origin.
2. Whether the peristalsis of the intestine is increased or diminished by ergot. If increased, whether this be due to a centric or peripheral action of ergot.

3. Whether the cause of the contraction of the blood vessels in the omentum is central or peripheral.

4. Whether ergot produces a rise or fall of blood pressure. Whatever change occurs, is it due to an action on the heart and arteries or on the spinal cord.

5. The action of ergot on temperature.

The experiments are then described at length. Ergotole is injected in quantities varying from 0.25 G. G. to 1 C. C. and its active physiological effect being demonstrated in from 3 to 5 minutes, the spinal cord is destroyed by means of a white hot wire, after which injections of even 2 C. C. are productive of no results, while electrical stimulation will still produce energetic uterine contractions.

(See Phila. *Med. News*, Jan. 31, 1891. pp. 133 to 139).

From these experiments he deduces "that ergot in producing contractions of the uterus, acts primarily and essentially upon the lumbar cord, i. e., its action in causing peristalsis of the uterus is centric, not peripheral."

His experiments with *ergotole* to establish whether the drug causes intestinal peristalsis by local action or by exerting an influence upon the spinal cord, he formulates by stating that, "It is justifiable, therefore, to conclude that ergot, in producing intestinal peristalsis, acts directly on the cord, and only reflexly upon the intestines, its action in this case, too, being centric, not peripheral."

From his experiments with *ergotole* upon the contractions of the arterioles he concludes "that ergot produces constriction of the arterioles and capillaries in the omentum and ear of rabbits and in the frog's web as long as the cord and the vagi are intact. These being destroyed, constriction is no longer produced by the drug; its action in this case is centric, not peripheral."

He proceeds now to employ *ergotole* in the investigation of its effect upon the blood pressure, and makes the following deductions :

1st. Ergot reduces the number of pulse-beats per minute.

2d. In the isolated frog's heart it reduces the force of the contractions.

3d. It exerts a local poisonous influence on the heart of the batrachian, as well as on that of the animal when injected into the jugular vein.

4th. Its main action, however, is exercised through the influence of the central nervous system.

5th. It raises arterial pressure when injected into the jugular vein of animals. The rise is preceded by a primary depression due to the local action on the heart.

6th. It is impossible at present to decide whether this local action is due to an influence on the heart, muscle, or on the cardiac ganglia.

Further experiments demonstrated that ergot causes a fall in temperature.

(See Phila. *Med. News*, 7th Feb. 1891, pp. 152–158).

The author next proceeds to a clinical study of ergot, and states that, "the therapeutic effects of few drugs correspond so closely with their physiological action as do those of ergot."

Upon the power of ergotole to constrict the arterioles and to cause arterial and capillary anaemia, depends its application in a large number of diseased conditions. It is of value in all haemorrhages. It has been successfully used in haemoptysis, haematemesis, epistaxis, haemorrhage from the gums, renal, haemorrhoidal and vesical haemorrhages; in the bleeding caused by carcinoma; and in the haemorrhage dependent upon a dyscrasis, as purpura haemorrhagica. In menorrhagia and leucorrhoea, which are produced by endometrial congestion. Ergotol is found of value in the colliquative sweating due to relaxation of cutaneous capillaries; in enlargement of the spleen, and in vascular goitre. It has been used in the successful treatment of aneurisms of various arteries.

Ergotole has also been advantageously used in congestive headaches and in impaired vision from congestion of the retina incident to dilated or hypertrophied heart. Intestinal catarrh with diarrhoea, due to congestion of the intestinal mucous membrane, is a special indication for the use of ergotole. In cases of dysentery, which proved rebellious to treatment with astringents, bismuth, opiates and even flushing of the large intestine, recovery occurred when the drug was persistently used. Its action was more immediate and more lasting when the ergotole was injected through a long rectal tube, than when given by the mouth or hypodermically. It has also been successfully used in chronic diarrhoea.

Ergotole has also a place in the therapy of certain affections of the heart, as in aortic insufficiency and so-called idiopathic dilatation of the heart; also in cases of arterio-schlerosis.

The writer has no new applications for the drug in labor, but states its applicability under the rules collated by Kehler, and given in most standard works.

Schatz, in his paper, on the use of ergot, contributed to the Third German Gynecological Congress, at Freiburg, concludes that the contractions of the uterus produced by ergot do not differ from the normal. That the action of ergot begins 15 minutes after its administration by the mouth and is greatest in 30 minutes, hence, it should not be given oftener than once in the hour. Small doses should be given to avoid tetanic contractions, which are an evidence of toxic action.

In gynecology, ergotole has been used in removing uterine fibroids by strangulating their vascular supply and by causing uterine contractions. It has been of benefit in cervicitis, in gonorrhoea, in paralysis of the bladder from distension, or in that due to cerebral or spinal lesion.

The writer has used ergotole with great success in cases of pneumonia and bronchitis. A chart is given with the record of the pulse-rate and temperature showing both to decline rapidly under the use of xv. gtt, of ergotole given every three hours. Cases are also alluded to, treated by Dr. N. S. Davis, who treated them successfully with this drug, sometimes in connection with digitalis.

We believe that ergotole exercises a very decided effect upon the pulmonary vessels.

Transudation has been proved by a very large number of observers, to depend upon the permeability and elastic distensibility of the blood vessels.

From these facts, we cannot fail to realize what a powerful agent this drug is in checking inflammatory exudation, as clinical experience has undoubtedly proven it to be in the first stage of pneumonia. If transudation depends upon the permeability and elastic distensibility of the vessels, we know that ergotole, by constricting these, can reduce perivascular engorgement.

If transudation is associated with increased heart's action we know that ergotole reduces the number of heart beats.

If the beginning of pneumonia exudation is associated with hurried breathing, we know that ergotole reduces the number of respirations.

If transudation is connected with fever, we know that ergotole reduces temperature.

If the fever in inflamatory exudations lowers blood pressure, we know that ergotole raises it.

All these physiological effects directly counteract the main features of the pathological process, and check further transudation, while the lymphatics carry away the exudation that has already occurred.

Ergotole may be advantageously used in certain cases of epilepsy, cases of grand mal, who complain of hemicrania during the intervals of rest. Here an opthalmoscopic comparison of the fundi oculorum should be made and if congestion is evident in one or both, ergotole should be tried. Among the symtpoms that should direct attention to possible cerebal congestion contracted pupil, hemiopia and diplopia, supraorbital pain and narrowing of the field of vision, and aggravation of the symptoms by the recumbent posture and acts involving deep inspiration, as blowing, sneezing, etc.

Ergotole is found efficacious in cases of nervous disease when the symptoms indicate hyperæmia and congestion of the spinal or cranial meninges, or an acute inflammation of the cord-substance.

In the treatment of the psychoses, in which ophthalmoscopic examination justifies us in diagnosticating intra-cranial congestion, and perhaps, inflammation, ergotole has proved to be a valuable adjunct to other means of treatment.

